# Stories of being a new nurse academic at a nursing education institution

**DOI:** 10.1002/nop2.1601

**Published:** 2023-01-09

**Authors:** Linda Mutenga, Charlené Downing, Irene J. Kearns

**Affiliations:** ^1^ Department of Nursing University of Johannesburg Johannesburg South Africa

**Keywords:** best moments, new nurse academic, nursing education institution, qualitative and appreciative inquiry

## Abstract

**Objectives:**

The objectives of this study were to explore and discover the best moments of being a new nurse academic at nursing education institutions.

**Aim:**

The purpose of this study was to define, discover and learn about the experiences of being a new nurse academic.

**Design:**

A qualitative design with inspiration from Appreciative Inquiry (AI) was used.

**Method:**

Purposive and snowball sampling was used. Data collection was done using semi‐structured individual interviews. Data were analysed using Giorgi's method. The COREQ guidance was used for reporting of this study.

**Results:**

Four themes were identified. (1) The emphasis was their transition from clinical setting into the world of academics, which was challenging, overwhelming and exciting all at the same time. (2) Participants were fulfilled and influenced to achieve more. (3) Participants expressed what they wished for. (4) Participants shared recommendations for job security, employment and retainment of new nurse academics.

## 1 INTRODUCTION

Nursing education and training plays a statistically significant role in the health profession of South Africa. This is evident as statistics show that the nursing industry is fast growing in South Africa. In the last 10 years, there has been a 35% increase in Registered Nurses produced from different Nursing Education Institutions to serve the community. In South Africa a more statistically significant percentage of nursing students who undergo nursing education and training prefers to be in a clinical setting upon completion; only a tiny percentage join the nursing education and training stream. There is now overwhelming evidence attesting to what many academics have known for years: academia is a highly stressful occupation. In fact, academics throughout the world deal with substantial ongoing occupational stress.

## 2 BACKGROUND

### 2.1 Nursing

Nursing represents the largest occupation in the healthcare sector, accounting for 59% of healthcare professionals (World Health Organization (WHO), 2020). Evidence shows that this has always been a trend. Kurtzman et al. (2010) state that nurses represent the single largest group of healthcare providers. Rispel (2015) agrees that South Africa and globally nurses make up the largest group of health service providers. Nurses are the key personnel in healthcare delivery, and they play a critical role Oldland et al. (2020). Professional nurses play different roles in the nursing profession, one of these roles being a nurse academic. Rispel (2015) says the nurse's role in nursing education is crucial and goes undisputed. The South African Nursing Council describes a nurse academic as an expert at a higher education institution, responsible for educating and training students (South African Nursing Council, 2021).

### 2.2 Higher education and its challenges

South African higher education has undergone a great transformation, which has caused a statistically significant increase in the job demands of academics (South African Government, 2012).Currently, many higher education institutions are recruiting new academics to join the field of teaching, research and community service, but they lack strategies to retain them (Lesenyeho et al., 2018). Evidence attests to what many academics have known for years: academia is highly stressful. Academics throughout the world deal with a statistically significant amount of ongoing occupational stress (Barkhuizen & Rothmann, 2016), because they are expected to venture in for more roles and responsibilities such as teaching, research and community service (Ibrahim et al., 2013:11). Robyn & Du Preez, 2013 and Tian and Lu (2017) state that new academics also face challenges when starting their academic journey.

### 2.3 New academics

The young nurse academics defined themselves as trendy. Being trendy helped the young nurse academics to adjust to the Covid19 challenge. Technology in health care is gaining ground at a breath‐taking pace. It is fast becoming an important part of future endeavours (Grady & Hinshaw, 2017:14). Van Houwelingen, Ettema, Kort and Ten Cate (2017:717) also suggest that specific competencies for technology have become important in nursing education and the young generation knows no world without technology. The young generation nursing students want exciting, engaging, technologically advanced and visually based form of learning (Chicca & Shellenbarger, 2018:181). To the new nurse academics, the new online teaching was somehow a continuation of what they had already started with their students. During this period the participants were made part of a driving change.

### 2.4 Generational gap

Watson (1994) states that a humanistic–altruistic value system begins early in life but continues to be influenced through interactions with friends, colleagues and nurse academics. When the new nurse academics enter into academia, they had expectations that the older generation of nurse academics would be more welcoming, but instead, they expressed their feelings of how difficult it was at first to work with them. Studies show that new nurse academics expressed disappointment or frustration about the older generation's lack of guidance and communication (Cangelosi, 2014). The new nurse academics also feel neglected and left without any guidance. They have to find their own strategies to cope. There is no support or any form of mentorship (Van Jaarsveldt, 2010).

New nurse academics are entering academia but, with the new generation rising, there is a loss of expertise that the older generation of nurses possesses (Price & Reichert, 2017). Therefore, the inevitable challenge is how the higher education institutions will augment the loss of retiring staff and their wealth of knowledge and experience whilst developing the newer academics (Tucker, 2020). Currently, in South Africa, nurses and nurse academic experts are nearing or at retirement age. As of 2020, the SANC statistics show that 17% of Registered Nurses are between 50–69 years of age (South African Nursing Council, 2020). This gives rise to a generational gap, which puts a question to mind: “Who will take over the role?” New nurse academics enter the academic world to perform and bring in new dynamics and challenges. The current challenges and notable gap prompted the researcher to want to understand what challenges the new nurse academics face and what can be done about these challenges. Therefore, the researcher asks the question: “What are the best moments of being a new nurse academic at a higher education institution?”

## 3 METHODS

To fully explore, understand and describe the phenomenon under study, the researcher used a qualitative, exploratory, descriptive and contextual research design.

The 5D's of the Appreciative Inquiry were used as open‐ended questions to gather detailed and well thought information from the participants. These questions gave the participants the comfort of expressing themselves openly, describe and give meaning to the phenomenon at study in their own words. The use of these questions allowed the researcher to get similar data from all the seven participants (Holloway & Galvin, 2017:90).

Using the 5D's of Appreciative Inquiry to formulate questions led the new nurse academics to discover their best moments amidst the challenges and led them to the realisation that they do enjoy being academics. A few of their best moments were when they realised how much they have already achieved and how much more they wanted to do, in addition to what they can offer the environment. There was a realisation of great enjoyment, finding of comfort zone and affirmation that academia is where they would like to be. The use of Appreciative Inquiry also led to an expression of great positivity and great hope and aspirations for the future.

The inclusion criteria of the study was as follows: Participants were new nurse academics, new does not refer to age but the number of years they have as new nurse academics. For this study, the new nurse academics were in this field for 1–5 years. Participants were Registered Professional nurses, registered with SANC under the terms of the Nursing Act. Participants had a BCur and master's in nursing qualification or were busy with a master's in nursing. Participants all worked in one of the nursing education institutions in South Africa at the time the study was conducted.

### 3.1 Study setting and sampling

The research study was conducted in two environments. In total, seven semi‐structured interviews were conducted. Four of these individual semi‐structured interviews were conducted at the participants' workplace, and three of the interviews were conducted in the participants' homes via Zoom. The different environments where the interviews were conducted were chosen by the participants. This allowed them to be comfortable. Of the four interviews, the researcher conducted two of these interviews in a boardroom in the Department of Nursing at one of these respective institutions. Two of the interviews were conducted at the participants' offices. The researcher travelled a journey of 105 km, which took 1 h 10 min to meet these participants at their respective institutions. Three of the interviews were conducted via Zoom. One Zoom interview was conducted with the participant from the Gauteng province, and the other two Zoom interviews were conducted with participants based in the Western Cape. All three participants were in the comfort of their homes as these were now their workspaces due to the pandemic. The different settings were the preference of the participants. In this study, purposive sampling was initially used. The researcher selected participants that had knowledge and understanding of the phenomenon being studied. The researcher was then referred to other new nurse academics in different nursing education institutions. Therefore, network (Snowball) sampling was also used. The final number of participants was 7. This was determined at the point when data saturation had been reached. Data saturation is when new data becomes redundant, and no new themes can be identified (Gray et al., 2017). The researcher found no new data from the participants. Data Saturation was confirmed by an independent coder.

### 3.2 Participants' demographic characteristics

The study consisted of three females and four males. Globally nursing has been dominated by females, in this study there is an overrepresentation of male nurses, this was due to availability and willingness to participate in the study. The researcher did not focus on gender inequalities. The ages ranged from 27 and 38 years. Five participants were from two cities from the Northeast region of South Africa and two participants were from the Southwestern Coast of South. All seven participants were black Africans.

### 3.3 Data collection

The researcher conducted a pilot study with one participant who was also a new nurse academic to confirm the adequacy of the interview questions in providing data on the phenomenon under study. The interview questions were not changed and the pilot study was made part of the main study. The researcher did data collection by means of conducting individual semi‐structured interviews until data saturation was reached. The semi‐structured interviews were audio‐recorded and of a duration of 45–60 min. Field notes were recorded immediately after the interviews had taken place and described the emotional context of the interviews and relevant observations, behaviours, reflections and experiences (Gray et al., 2017). Member checking was used, thereby verifying with the participants the researcher's understanding of the observations and asking the participants to provide oral comments on the field notes. Data collection took place over the period of March 2020 until June 2020.

### 3.4 Interview questions and description of appreciative inquiry phases.

- How would you define being a new nurse academic? [Definition stage].

During this stage, the participants were given an opportunity to define in their own terms and understand what a nurse academic meant to them being.

- How would you describe your best moments as a new nurse academic? [Discovery stage]

The discovery stage allows participants to discover what works well (Trajkovski et al., 2015). During this stage, the participants were describing what moments felt like the best moments to them.

- If you look at your institution, what would you like to see as a new nurse academic? [Dream stage]

During this stage, the participants were allowed to have their wildest dreams in terms of what they would love to have in their institutions.

- What needs to be designed to provide the best moments for new nurse academics? [Design stage]

This phase focuses on designing what should be. Each participant was asked to design what could be an ideal environment to have the best moments (Trajkovski et al., 2015).

- What can be recommended, developed and maintained to ensure the best moments in your institution? [Destiny stage]

The destiny phase focused on how the envisioned future would be sustained and deciding what needed to happen to ensure the best moments (Trajkovski et al., 2015). Participants were asked to provide their recommendations for their respective institutions.

### 3.5 Data analysis

The researcher collected, transcribed and analysed the data using Giorgi's five‐step method to obtain the essence of the participants' experiences (Giorgi, 2009). The researcher transcribed the recorded interviews verbatim. The researcher read and reread the entire written account to make sense of the whole phenomenon. Thereafter “units of meaning” were recognised and categorised (Giorgi, 2009). The researcher analysed data, and an independent coder and the supervisors were also involved in the consensus. The independent coder was an individual with a PhD in nursing and extensive experience in qualitative research. All data were labelled according to the date of collection. The transcriptions and consensus conversations included field notes, and participants' gestures included non‐verbal and verbal communication (Miles et al., 2018).

### 3.6 Trustworthiness

The trustworthiness of this study was assessed by using the four criteria of credibility, transferability, dependability and confirmability (Korstjen & Moser, 2018). During this study, credibility was demonstrated by prolonged engagement as the researcher spent sufficient time with participants (Korstjen & Moser, 2018). During this time, rapport was established, and a deeper understanding of being a new nurse academic was gained. During the in‐depth, semi‐structured individual interviews, the researcher focused on the participants' behaviours, expressions and how they expressed their best moments. Purposive sampling was used to ensure that the phenomena under study were fully explored. There was a detailed description of the study results to achieve transferability and an audit trail of all research activities to enhance dependability and confirmability. Furthermore, the researcher consulted an independent coder to ensure the trustworthiness of the study by checking and confirming that accurate units of meaning were derived from the collected data.

### 3.7 Ethical considerations

Before the commencement of this study, permission was requested, and approval was obtained from the Faculty of Health Sciences Research Ethics Committee (REC‐110‐2019) and Higher Degrees Committees (HDC 01‐54‐2019) of the university of (Johannesburg). Permission from the gatekeepers from the relevant higher education institutions was also granted. Ethical considerations were observed as laid down by the ethics for medical research. The researcher adhered to the following ethical principles: respect for autonomy, beneficence, non‐maleficence and justice (Dhai & McQuoid‐Mason, 2011). As data was also collected via a technology platform, the researcher adhered to strict ethical principles about the privacy of information (Holloway & Galvin, 2017).

Emails were sent out to the participants to request their participation in the study. The researcher acted in the best interest of all the participants. The participants, therefore, were seen as autonomous individuals, having the right to decide voluntarily to participate in the study without the risk of discriminatory treatment or coercion. Participants gave written consent, and confidentiality and anonymity were assured as, during coding, the interviews were labelled with numbers. For example, “participant 1” were used. Assurance was given to the participants that they could withdraw from the study at any time without being penalised in any way.

## 4 RESULTS

### 4.1 Emerging themes

Four themes and nine categories were identified from the in‐depth individual semi‐structured interviews. The themes and categories developed from the interviews with participants in accordance with the questions asked. The themes were as follows: (1): Defining being a new nurse academic: The emphasis was their transition from clinical setting into the world of academics, which was challenging, overwhelming and exciting all at the same time. (2): The best moments of being a new nurse academic. Participants were fulfilled and influenced to achieve more. (3): The dream of being a new nurse academic: Participants had wishes/ideals for their respective institutions. (4): Designing best moments for new nurse academics. Participants shared recommendations of job security, employment and retainment of new nurse academics.

### 4.2 Defining being a new nurse academic

The participants discussed how they defined themselves as being new nurse academics. The participants elicited that they felt privileged being new nurse academics. There were a variety of feelings ranging from being grateful for the opportunity, empowered, excited, eager, proud and hopeful. The new nurse academics expressed how this beautiful journey of transitioning from clinical nursing practice to academia was also embedded with great challenges. These challenges included being overwhelmed, intimidated, fearful, uncertain and disappointed.

#### 4.2.1 Being a new nurse academic is a great challenge and a great privilege

The participants ventured into telling their story about being a new academic, with specific reference to the challenges. The emphasis of the sharing was their transition from clinical setting into the world of academics, which was challenging, overwhelming and exciting all at the same time.

I think being a new nurse academic has a kind of an oxymoron effect, bittersweet because, at the same, it's a huge opportunity that people my age can really ever get. At the same time, it is challenging, and it is not easy. There are a lot of things that we face on a daily basis. (Participant 4, Male, Age 32).

“Although there were challenges, I also think that the opportunities far surpass the challenges.” (Participant 4, Male, Age 32).

#### 4.2.2 The new nurse academics are new and trendy but experience a generational gap between themselves and the older academic generation

The new nurse academics defined themselves as trendy. “Like we are new, we can use so much of technology to make the classroom a vibrant environment.” (Participant 1, Female, Age 27). The participants had expectations that the older generation of nurse academics would be more welcoming, but instead, they expressed their feelings of how difficult it was at first to work with them. “I found it difficult to work with people from the previous generation.” (Participant 3, Male, Age 29).

### 4.3 The best moments of being a new nurse academic

The participants described their best moments in a variety of ways. They described their best moments as the ability to deliver what was expected of them and the privilege of being exposed to research and presentations at national and international conferences. The best moments provided the participants with an assurance that they wished to stay in academia.

#### 4.3.1 Fulfilment in delivering what was expected of them as new nurse academics

During the interviews, the participants mentioned how lecturing was the best moment for them. “I get the best of joy when I'm in class standing in front of students.” (Participant 4, Male, Age 32). One participant expressed how this Fulfilment was a continuous feeling and excitedly said: “I feel like it's an everyday fulfilment.” (Participant 5, Female, Age 38).

#### 4.3.2 Exposure to nursing research, national and international conferences

The participants expressed how nursing research played a statistically significant role in their journey of being new nurse academics and how it gave them confidence in academia. Due to nursing research, they have been exposed to national and international conferences. Expressing one of best moments, one participant said, “One of my best moments is the chance to actually be involved in nursing research.” (Participant 2, Male, Age 32). Full of excitement, one participant shared his experience of attending conferences and said, “I still remember my first conference.” The participant continued to share the confidence they gained from attending conferences by saying: “And I believe that those conferences, they built me to be the person that I am today, with confidence. I now have confidence in everything that I do.” (Participant 3, Male, Age 29).

Their best moments made them feel great as new nurse academics. With a high‐pitched voice, one participant put so much emphasis and said, “I feel great!”*…* “I feel very great, I feel honoured”*…* “It feels great!” (Participant 5, Female, Age 38). The positive feedback from the students and colleagues was also an affirmation that they were meeting the expectations.

### 4.4 The dream of being a new nurse academic

#### 4.4.1 The need for moral support and a formal mentorship program

Discussing their dreams as new nurse academics, the participants expressed their wishes for their perspective institutions. Their dreams included the need for moral support from experienced colleagues. The new nurse academics expressed how they felt alone and uncared for. They were left to their own devices in academia. The participants verbalised how there was a lack of mentorship, and they really wished they could be mentored. Speaking about mentorship, one participant expressed her views: “I wish there were like a formal process in the university. Like for new academics “nje” (only) to… to like mentor you.” “So instead of reminding me all the time how inexperienced I am… like… why don't you take my hand and show me the ropes?” (Participant 1, Female, Age 27). One participant then said something very profound in closing this matter: “I want to be moulded into what I want to become”… “Yes. A better version of myself, allow me to grow.” (Participant 2, Male, Age 32).

#### 4.4.2 The participants longed for continued exposure to the clinical field

The participants showed interest in being in the clinical field on an arrangement basis so that they could be aware of changes in the clinical field. The new nurse academics showed that they were aware of the gap that existed between nursing theory and clinical practice. Showing disappointment on this matter, one participant said: “We become so disconnected from the clinical field! …” (Participant 1, Female, Age 27). Showing the benefits of such clinical exposure, another participant said: “When we go to the hospital, we can deal with the cognitive part, the psychometric and affective part of learning.” Deliberating on the relevance of this matter, one participant expressed this with pride: “For me to stay relevant, it means I need to work with the students one day of the week so that I can stay relevant, and I can explain to the students what is happening now.” (Participant 7, Male, Age 36).

### 4.5 Designing best moments for new nurse academics

#### 4.5.1 The new nurse academics requested to attend structured programmes to learn skills

As the interviews continued, the participants expressed their views on how their respective institutions could create the best moments for new nurse academics. These include programmes to prepare the participants for the needed skills in academia and a succession plan where experience and knowledge should be shared. About the skills the new nurse academics wished to be taught and trained in specific things like presenting a class, leadership, and teaching methodologies, one participant said: “We are doing a generic orientation …” (Participant 7, Male, Age 36). Expressing this, the participant shared her wishes, “I wish there was like a formal process in the university like for new academics.” (Participant 1, Female, Age 27).

#### 4.5.2 The participants needed a succession plan between the old and new generations

Globally, older generation of nurses represent exceedingly skilled and experienced group in the healthcare system (Markowski et al., 2020). With the experienced older generation of nurse academics retiring, the young nurse academics may still be lacking the expertise and may still be unfamiliar with the expectations and responsibilities of the academic role (Brown & Sorrell, 2017). Succession planning has not been a common practice in nursing, but now discussion about this strategy, are becoming more visible (Tucker, 2020). During the interviews, the participants also focused on how the older generation should transfer knowledge to the new academics in a structured way. One participant shared the need for a succession plan: “Succession plan yes, the ability to transfer skill onto the newer generation …” (Participant 7, Male, Age 36). Expressing the need for succession plans, another participant said: “Like if I feel like I'm capacitated to do the job, I won't be frustrated doing it.” (Participant 1, Female, Age 27). Succession planning is a strategy that will allow the nursing education institutions to manage environmental challenges effectively. (Phillips et al., 2017).

#### 4.5.3 The participants wished for job security and being retained as new nurse academics and wanted to see an increase in the employment of more new nurse academics

The participants recommended that newer academics should be employed. About this, one participant said: “More new nurse academics are needed, and they should be brought in…” (Participant 5, Female, 38). Another participant also verbalised how the institutions do not retain new nurse academics: “Institutions should start investing, actually they are investing, but they should start retaining us. They are grooming people and letting them go. …” (Participant 3, Male, Age 29).

## 5 DISCUSSION

### 5.1 Theories used in the study

In this study, the researcher was guided by the philosophy of qualitative research, which is interpretive, humanistic and naturalistic (Creswell, 2007). It places statistically significant importance on subjectivity. One of the key components of qualitative philosophy is ontology. Ontology deals with nature or involves the philosophy of reality. The ontological assumption is that there is no single reality but encompasses multiple realities for any phenomenon (Speziale & Carpenter, 2003). Kaushik and Walsh (2019:2) state that in an ontological assumption, the researcher relies extensively on the views of the participants and thereafter develops subjective meanings of the phenomena under study. The young nurse academics constructed their own realities in their respective nursing education institutions. The participants could construct their own reality by dreaming and designing ideas and structures that would elicit best moments, leading to a great destiny for themselves according to principles of appreciative inquiry. The researcher also incorporated the Watson's caring theory. Watson's theory promotes caring as a core of the nursing body. Watson (2008) Theory of Human Caring is widely used in nursing practice and contemplates that nurses' humanity embraces the humanity of others to preserve dignity of self and others (Watson, 2012:8). Watson also defines caring moments as heart‐centred encounters with another person. In the study, the caring moments were measured by how the new nurse academics experienced best moments. Watson's carative factors were also incorporated in the study. Carative factors are deeper and larger dimensions of nursing that go beyond the changing times, setting, procedures, functional tasks, specialised focus around disease, treatment and technology (Watson, 2008). There were also recommendations formed based on these carative factors.

### 5.2 Transition and challenges of being a new nurse academic

The study findings showed that the new nurse academics were challenged initially, but they shared that they were present in a great space. As they told their experiences and reflected on these, they recognised their growth and ability to overcome the adversity they faced. They paved their own way and realised their strengths and tremendous growth as new nurse academics, and this gave them resilience. Hill and MacGregor (1998) show that any transition process has three main phases: challenge, confusion and adaptation. Dolan Hunt (2018) concurs that the academic role is rewarding and yet full of challenges. The demands associated with academic life have created considerable new challenges for the new nurse academics (Halcomb et al., 2016). McDermid et al. (2018) and Legare and Armstrong (2017) confirm that the transition from the clinical setting to the academic environment has been proven to be a statistically significant challenge. The transition was, for most of the participants, a very lonely journey. They needed someone to hold their hand (Wyllie et al., 2019).

### 5.3 The generational gap in nursing

Ryan et al. (2017) agree that the global nursing workforce is ageing. These older generation nurse academics play a key role in the preparation of the next generation nurses. Although the new nurse academics felt intimidated by the older generation, they found common ground to work with them and trust them. The new generation had to form relationships with the older and experienced generation to repair relationships. By forming relationships with the older generation, the new nurse academics eventually felt a sense of belonging. This was important for learning, growth and workplace satisfaction (Wyllie et al., 2019).

### 5.4 Best moments experienced by the new nurse academics

The new nurse academics revealed that they had the best moments in their respective institutions. These best moments were a result of knowing what to do and being confident. The participants shared how they looked forward to lecturing. They had a commitment towards their students (Ashraf et al., 2017). Lecturing behaviour plays a pivotal role in the linkage between lecturers' competence and students' satisfaction with lecturing (Opatha, 2020). The participants expressed how nursing research played a statistically significant role in their journey of being new nurse academics and how it gave them confidence in academia. Due to nursing research, they have been exposed to national and international conferences. Research informs best practice in nursing (Edward, 2015). The best practice is continuously changing in the field of nursing (Chinn & Kramer, 2017). Edward (2015) states that nurse academics' involvement, confidence and engagement in research are an area for continued growth.

### 5.5 Mentorship

The participants had a dream that there would be policies in place about formal mentorship programmes in their respective nursing education institutions. Dolan Hunt (2018) claims that mentoring can positively influence the development of self and career. Dolan Hunt (2018) and McArthur‐Rouse (2008) agree that a mentor assists the new nurse academic to have a smoother transition into academia. Providing moral support and mentoring can be viewed as a form of caring. McArthur‐Rouse (2008) concurs that mentoring new nurse academics is a successful strategy to promote and nurture a collegial and caring environment.

### 5.6 Appropriate skills needed by the new nurse academics

Globally, concerns have been raised about the lack of formal training and inadequate preparation for new nurse academics (Summers, 2017). Nesse (2003) argues that “clinical expertise alone does not qualify one to be an academic.” A nurse academic should be involved in clinical practice and education (Bullin, 2018). Booth et al. (2016) agree that new nurse academics at nursing education institutions need to possess knowledge and skill in pedagogical practice and the clinical practice area in which they teach. The nursing education institutions need to expose the new nurse academics early to appropriate skills and career developing opportunities (Singh et al., 2019). The participants showed their longing to be involved in the clinical field.

### 5.7 Recruitment and job security

The new nurse academics expressed how they need to have job security. The recruitment and retention of appropriately qualified academics are vital to the delivery of nursing programmes (McArthur‐Rouse, 2008). Tucker (2020) advises that nursing education institutions need to plan for the future by recruiting, developing and retaining nurse academics. Institutions must invest in resource building like offering salary benefits and permanent employment to retain their new nurse academics. Brown and Sorrell (2017) state that if these new nurse academics do not feel secure in their positions, they may leave the higher education institutions. McArthur‐Rouse (2008) continues to say that higher education institutions need to recruit new academics and retain and develop them. This will assist the new nurse academics to provide the relevant expertise.

### 5.8 Empirical and theoretical contributions

The study contributed to the possibilities of new nurse academics having best moments in higher education institutions. It proved that positivity as mentioned in Appreciative Inquiry can bring about hard work in an organisation. Appreciative Inquiry involves the art and practice of asking questions that strengthen a system's capacity to heighten positive potential. The authors used the 5D's of Appreciative Inquiry as the main questions in order to elicit positivity (best moments) that the new nurse academics have experienced in their institutions.

#### 5.8.1 The gap

Current research centres does not show the outcomes of appreciative inquiry used in academia. Future research is anticipated to close this gap by, if possible, describing best moments of new academics.

### 5.9 Limitations of the study

This study only focused on nursing academics based in Gauteng and the Western Cape. It was limited to the new nurse academics in the nursing education institutions in the university setting and not the nursing colleges. It only focused on new academics who were nurses and not new academics in other professions. It was limited to the views of new nurse academics, referring to those that have been in academia for 1–5 years. The views of those who are in the mid‐career were not considered. It only focused on the new nurse academics generation and not the older nurse academics generation. The study did not include all ethnic groups and there was no focus on barriers based on gender.

### 5.10 Recommendations

- Recommendations for the higher education

Institutional policies about employment and retainment of new nurse academics should be revised and implemented to ensure that the young nurse academics have job security.

- Recommendations for nursing management

To support the new nurse academics, the head of the nursing departments who act as managers need to inform them of the counselling services that the nursing education institutions provide, so that they may express their challenges.

- Recommendations for further research

Further study on the experiences of young academics is needed to improve retention and retainment.

- Recommendations based on the carative factors by Watson, that were used in the study

The new nurse academics incorporated the carative factors to show caring to themselves, their colleagues and students.

## 6 CONCLUSION

The study showed the need for guidance, support and mentorship in the transition of becoming a new nurse academic. Without formal and structured mentorship programmes, the new nurse academics will always experience challenges as they begin their journey in academia. The higher education institutions need new and fresh nurse academics, but they need to device policies to retain them. These include, mentorship, training programmes, exposure to nursing research and job security. Although the new nurse academics had challenges in the beginning of their journey, they verbalised that they found their feet and eventually had best moments, and these enabled them to stay in academia. The use of appreciative inquiry also enabled the new nurse academics to not only see the challenges but to also see the positivity that led to best moments. Perhaps the use of appreciative inquiry should increase in the nursing fraternity. Watson's theory was used to put emphasis on the fact that nursing is guided by caring.

## AUTHOR CONTRIBUTIONS

Study design: L.M., C.D. and I.J.K. Data collection: L.M. Data analysis: L.M., C.D. and I.J.K. Manuscript writing and revisions for important intellectual content: L.M. and C.D.

## FUNDING INFORMATON

No funding was received for the study.

## CONFLICT OF INTEREST

The authors declare no potential conflicts of interest in respect to the research, authorship and publication of this article.

## Data Availability

The authors elect not to share data.
